# Initial magnetic resonance imaging/magnetic resonance angiography may miss intracranial artery dissection in young adults with stroke: the role of subsequent imaging

**DOI:** 10.1093/esj/aakag115

**Published:** 2026-09-18

**Authors:** Naoki Makita, Tomoyuki Ohara, Masahiro Makino, Eijirou Tanaka, Daiki Fukunaga, Naoki Tokuda, Akihiro Fujii, Hidesato Takezawa, Keisuke Imai, Takehiro Yamada, Yoshinari Nagakane, Shiori Ogura, Keiko Maezono-Kandori, Toshiki Mizuno

**Affiliations:** Department of Neurology, Kyoto Okamoto Memorial Hospital, Kyoto, Japan; Department of Neurology, Kyoto Prefectural University of Medicine, Kyoto, Japan; Department of Neurology, Kyoto Okamoto Memorial Hospital, Kyoto, Japan; Department of Neurology, Kyoto Prefectural University of Medicine, Kyoto, Japan; Department of Neurology, Kyoto Prefectural University of Medicine, Kyoto, Japan; Department of Neurology, Kyoto Second Red Cross Hospital, Kyoto, Japan; Department of Neurology, Saiseikai Shigaken Hospital, Shiga, Japan; Department of Neurology, Saiseikai Shigaken Hospital, Shiga, Japan; Department of Neurology and Stroke, Kyoto First Red Cross Hospital, Kyoto, Japan; Department of Neurology and Stroke, Kyoto First Red Cross Hospital, Kyoto, Japan; Department of Neurology, Kyoto Second Red Cross Hospital, Kyoto, Japan; Department of Neurology, Kyoto Second Red Cross Hospital, Kyoto, Japan; Department of Neurology, Kyoto Prefectural University of Medicine, Kyoto, Japan; Department of Neurology, Kyoto Prefectural University of Medicine, Kyoto, Japan

**Keywords:** cervicocephalic arterial dissection, ischaemic stroke, magnetic resonance angiography, vessel wall imaging

## Abstract

**Introduction:**

Intracranial artery dissection (IAD) is an important cause of ischaemic stroke in young adults; however, its diagnosis at initial presentation remains challenging. We aimed to determine how often initial magnetic resonance imaging (MRI)/magnetic resonance angiography (MRA) and subsequent imaging identified IAD, and to clarify the timing of IAD diagnosis.

**Methods:**

We performed a secondary analysis of a prospective, multicentre registry of 275 adults aged 18–50 years admitted with acute ischaemic stroke within 14 days of onset. All patients underwent MRI/MRA at admission, followed by scheduled follow-up MRI/MRA 7 days later. Vessel wall MRI (VW-MRI), digital subtraction angiography (DSA) and computed tomography angiography (CTA) were performed when clinically indicated. The proportion of patients in whom each examination identified IAD and the time to diagnosis were assessed.

**Results:**

IAD was diagnosed in 53 patients. Initial MRI/MRA identified IAD in only 9 (17%; 95% CI, 8–30). The median time to diagnosis was 5 days after admission; 77% had been diagnosed by Day 7, and the latest diagnosis was made on Day 35. Among patients who underwent each examination, the observed diagnostic yield was 38 of 45 for VW-MRI (84%; 95% CI, 71–94), 41 of 53 for follow-up MRI/MRA (77%; 95% CI, 64–88), 15 of 30 for DSA (50%; 95% CI, 31–69) and 7 of 34 for CTA (21%; 95% CI, 9–38).

**Conclusion:**

Among young adults with ischaemic stroke who ultimately met the imaging criteria for IAD, initial MRI/MRA alone was insufficient to establish the diagnosis in most cases. Repeated and multimodal imaging contributed to the diagnosis of IAD.

## Graphical Abstract

**Graphical Abstract:**
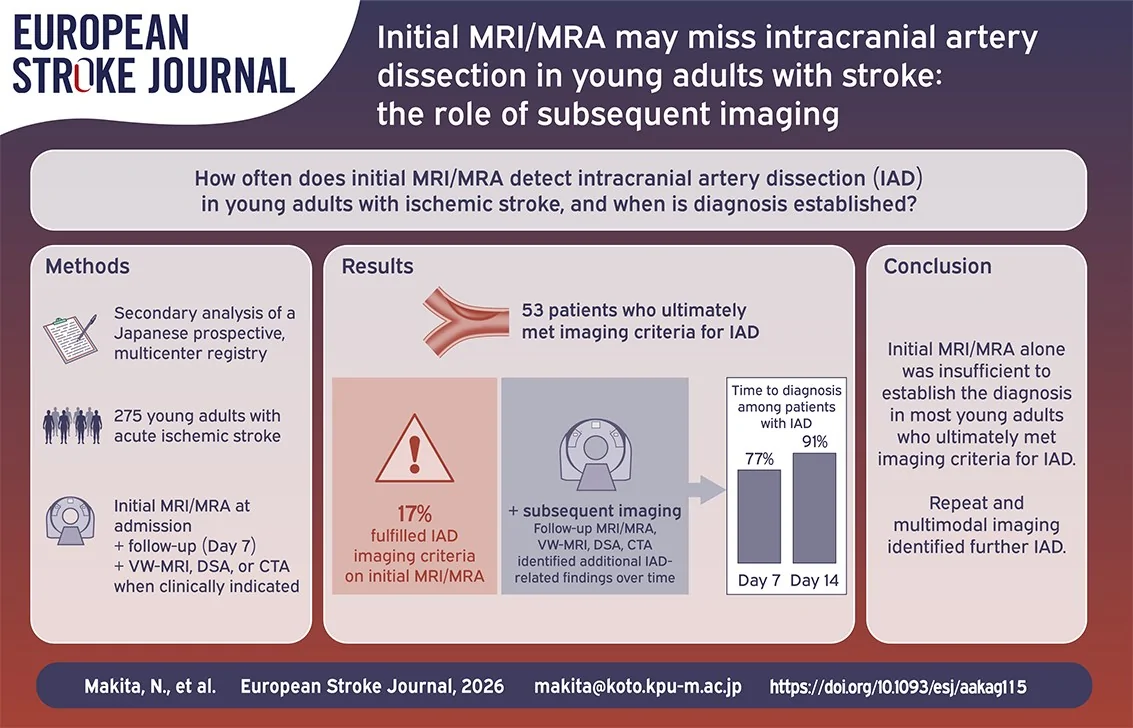

## Introduction

Cervicocephalic artery dissection is an important cause of ischaemic stroke in young adults, with notable regional differences in its anatomical distribution.^1^^,^^2^ While studies from Western countries have predominantly focused on extracranial artery dissection,^3^^,^^4^ previous reports showed that intracranial artery dissection (IAD) is more prevalent than extracranial dissection in East Asian populations.^5–7^ Accordingly, accurate identification of IAD is particularly important in the diagnostic evaluation of young stroke patients in East Asia.

Despite its clinical importance, the diagnosis of IAD at initial presentation remains challenging. In particular, magnetic resonance imaging (MRI), including magnetic resonance angiography (MRA), may fail to detect IAD in the acute phase. This limitation can be partly attributed to the dynamic morphological changes that occur during the course of arterial dissection. Typical imaging findings of IAD, such as intramural haematoma, double lumen and intimal flap, may be subtle or absent on early imaging and only become apparent on late imaging.^8^ Consequently, IAD may be overlooked on initial MRI/MRA.

Although several additional imaging approaches, including vessel wall MRI (VW-MRI), other vascular imaging techniques and follow-up MRI/MRA, have been reported to aid in the diagnosis of IAD,^9^^,^^10^ their respective contributions to establishing the diagnosis after an inconclusive initial MRI/MRA have not been systematically evaluated.

The aims of the present study were to determine how often initial MRI/MRA and subsequent imaging identified IAD, and to clarify the timing of IAD diagnosis, in young adults with ischaemic stroke.

## Methods

### Study design

This was a secondary analysis of the SKYSTER (Shiga Kyoto Young Stroke Evaluation Registry) study, a prospective, multicentre registry conducted in the Kyoto-Shiga region of Japan that enrolled 275 adults aged 18–50 years who were admitted with acute ischaemic stroke within 14 days of onset to 5 high-volume stroke centres (University Hospital of Kyoto Prefectural University of Medicine, Kyoto First Red Cross Hospital, Kyoto Second Red Cross Hospital, Kyoto Okamoto Memorial Hospital and Saiseikai Shigaken Hospital) from February 2018 to January 2023.^7^ Acute ischaemic stroke was defined as the presence of focal neurological symptoms together with acute ischaemic lesions confirmed on diffusion-weighted imaging (DWI). Stroke aetiology was classified according to the TOAST criteria based on the protocol-based workup, as described previously.^7^^,^^11^ IAD was diagnosed based on previously reported imaging diagnostic criteria and further classified into 3 categories: definite, probable or possible, as detailed in Table S1.^2^ After independent local evaluations at each participating institution, all imaging data were transferred to a central core laboratory for consensus review by 2 stroke neurologists experienced in cerebrovascular imaging. The 2 reviewers assessed whether the imaging findings of each examination fulfilled the diagnostic criteria and then determined the final diagnosis of IAD by consensus. These assessments were not blinded to clinical information, the timing of the imaging examination or findings from other imaging examinations.

### Data collection

The collected baseline data included sex, age, medical history (hypertension, diabetes mellitus, dyslipidaemia, migraine and smoking status), presenting symptoms (headache and neck pain), National Institutes of Health Stroke Scale (NIHSS) score at admission, time from onset and admission to diagnosis by each imaging examination, and time from onset and admission to each imaging examination. The day of admission was defined as Day 0. The imaging workup protocol included brain MRI/MRA at the baseline (Day 0), and 7 ± 3 days after admission (Days 7 ± 3). Digital subtraction angiography (DSA), computed tomography angiography (CTA) or VW-MRI was additionally performed at the discretion of the attending physician when clinically indicated.

### Imaging findings

Imaging findings, evaluated on MRI/MRA, VW-MRI, DSA and CTA, included intramural haematoma, intimal flap or double lumen, stenosis or occlusion of the intracranial artery secondary to development towards dilation, pearl-and-string sign, rapid change in morphology, aneurysmal dilation, stenosis, occlusion, location of IAD and location of ischaemic lesions. Intramural haematoma was defined as eccentric-bright elements in the arterial wall on VW-MRI or axial source images of MRA, with a signal intensity greater than 200% of that of the sternocleidomastoid muscle in the artery wall, often appearing as crescent-shaped thickening.^12^^,^^13^ A double lumen was defined as the presence of a false lumen or an intimal flap on VW-MRI, DSA or CTA. Aneurysmal dilation was defined as a vessel diameter of at least 1.5 times the width of a normal distal artery. The pearl-and-string sign was defined as the coexistence of aneurysmal dilation and the string sign (luminal narrowing of 30% or more).^14^ A rapid change in morphology was defined as any of the following findings on follow-up compared with initial MRI/MRA: (1) fusiform or irregular aneurysmal dilation at a non-branching site with increased or reduced size, or subsequent appearance of stenosis; or (2) long filiform or irregular stenosis with increased or reduced size, or subsequent appearance of aneurysmal dilation. The location of arterial dissection was classified as intracranial or extracranial based on MRA, CTA or DSA. For the vertebral artery (VA), an intracranial dissection was defined as the involvement of the V4 segment, distal to the point where the artery pierces the dura at the foramen magnum; for the internal carotid artery, it was defined as the segment distal to the distal dural ring at the level of the anterior clinoid process.

### MRI protocol

Initial and follow-up MRI/MRA were performed using a 3 T or 1.5 T scanner. The routine MRI/MRA protocol in our study included DWI with apparent diffusion coefficient maps, fluid-attenuated inversion recovery and 3D time-of-flight (TOF) MRA of the intracranial arteries. Cervical MRA was not included in the routine protocol. VW-MRI, when performed, was obtained using 3D T1-weighted black-blood imaging of the vessel wall on a 3 T or 1.5 T scanner. Imaging protocols varied across institutions and scanners.

### Institutional ethical approval

This study was approved by the Institutional Review Board of Kyoto Prefectural University of Medicine (approval no.: ERB-C-1024) and institutional review boards of the 4 other participating hospitals. The study was conducted in accordance with the Declaration of Helsinki, and all participants provided written informed consent before enrolment.

### Statistical analysis

Clinical characteristics were compared between patients with IAD (IAD group) and those whose ischaemic stroke was attributable to causes other than IAD (non-IAD group). Categorical variables underwent comparison using the Pearson chi-square test or Fisher’s exact test, as appropriate. Continuous variables were compared using the Mann–Whitney U test. The observed diagnostic yield of each imaging examination was defined as the proportion of examined patients whose imaging findings fulfilled the diagnostic criteria for IAD, with 95% CIs calculated using the Clopper-Pearson exact method. A sensitivity analysis restricted to patients with definite IAD was performed to assess the robustness of the main findings. All statistical analyses were performed using R version 4.4.1. A 2-sided *P*-value of <.05 was considered significant.

## Results

Of the 275 young patients with ischaemic stroke (median age, 46 years; 71% men; median NIHSS 2), 53 (19%; median age, 46 years; 40 men) were diagnosed with IAD (IAD group), and the remaining 222 had other causes (81%; non-IAD group). The distribution of stroke aetiologies in the whole cohort is provided in Table S2; extracranial artery dissection accounted for 15 patients (5%). The most frequently dissected artery was the intracranial VA (*n* = 23), followed by the middle cerebral artery (*n* = 9) (Table 1).

**Table 1:** Location of dissected artery.

Location	*n* = 53
**Single-vessel dissection**	
** VA**	23 (43)
** MCA**	9 (17)
** ACA**	7 (13)
** PICA**	5 (9)
** ICA**	2 (4)
** BA**	2 (4)
** PCA**	2 (4)
**Multi-vessel dissection**	
** ICA and MCA**	2 (4)
** ICA, MCA and ACA**	1 (2)

Data are presented as *n* (%).

Abbreviations: ACA = anterior cerebral artery; BA = basilar artery; ICA = internal carotid artery; MCA = middle cerebral artery; PCA = posterior cerebral artery; PICA = posterior inferior cerebellar artery; VA = vertebral artery.

Table 2 shows the baseline characteristics in IAD and non-IAD groups. The IAD group, on comparison with the non-IAD group, had a lower prevalence of diabetes mellitus (8% vs. 20%, *P* = .03), whereas headache (60% vs. 14%, *P* < .01) and neck pain (21% vs. 6%, *P* < .01) were significantly more common in the IAD group. Regarding infarct location, the IAD group, compared with the non-IAD group, showed a higher frequency of lesions in the medulla oblongata (23% vs. 5%, *P* < .01), but a lower frequency in the corona radiata (4% vs. 15%, *P* = .02).

**Table 2:** Baseline characteristics in IAD and non-IAD groups.

	IAD (*n* = 53)	Non-IAD (*n* = 222)	*P*-value
**Male**	40 (75)	155 (70)	.42
**Age**	46 [41, 48]	46 [42, 49]	.41
**Hypertension**	25 (47)	109 (49)	.80
**Diabetes mellitus**	4 (8)	44 (20)	.03
**Dyslipidaemia**	30 (57)	133 (60)	.66
**Current smoking**	26 (49)	121 (55)	.46
**Migraine**	8 (15)	23 (10)	.33
**Headache**	32 (60)	32 (14)	<.01
**Neck pain**	11 (21)	13 (6)	<.01
**NIHSS**	3 [1, 5]	2 [1, 5]	.52
**Location of infarct**			
**Multiple lesions**	20 (38)	86 (39)	.53
**Single lesion**			
** Cortical**	7 (13)	23 (10)	.62
** Subcortical**	2 (4)	7 (3)	.68
** Posterior limb of internal capsule**	0	13 (6)	.80
** Corona radiata**	2 (4)	34 (15)	.02
** Putamen**	0	5 (2)	.59
** Thalamus**	1 (2)	12 (5)	.47
** Midbrain**	0	6 (3)	.60
** Pontine**	3 (6)	12 (5)	1.00
** Medulla oblongata**	12 (23)	11 (5)	<.01
** Cerebellum**	4 (8)	12 (5)	.52

Data are presented as medians [interquartile range] for continuous variables or *n* (%) for categorical variables.

Mann–Whitney U test, Pearson’s chi-square test or Fisher’s exact test was performed, as appropriate.

Abbreviation: IAD = intracranial artery dissection.

Patients with IAD were classified into definite (*n* = 43), probable (*n* = 7), and possible (*n* = 3) cases according to the imaging diagnostic criteria. Initial MRI/MRA, follow-up MRI/MRA, VW-MRI, DSA and CTA were performed in 53 (100%), 53 (100%), 45 (85%), 30 (57%) and 34 (64%) patients, respectively. The initial MRI/MRA was performed at a median of 0 days after onset (interquartile range [IQR]: 0–1), and on the day of admission. The median time intervals from onset to follow-up MRI/MRA, VW-MRI, DSA and CTA were 7 days (IQR: 7–10), 7 days (IQR: 5–14), 4 days (IQR: 1–11) and 2 days (IQR: 1–8), respectively; the corresponding intervals from admission were 7 days (IQR: 6–9), 7 days (IQR: 3–11), 4 days (IQR: 1–11) and 2 days (IQR: 0–6), respectively.

**Figure 1:**
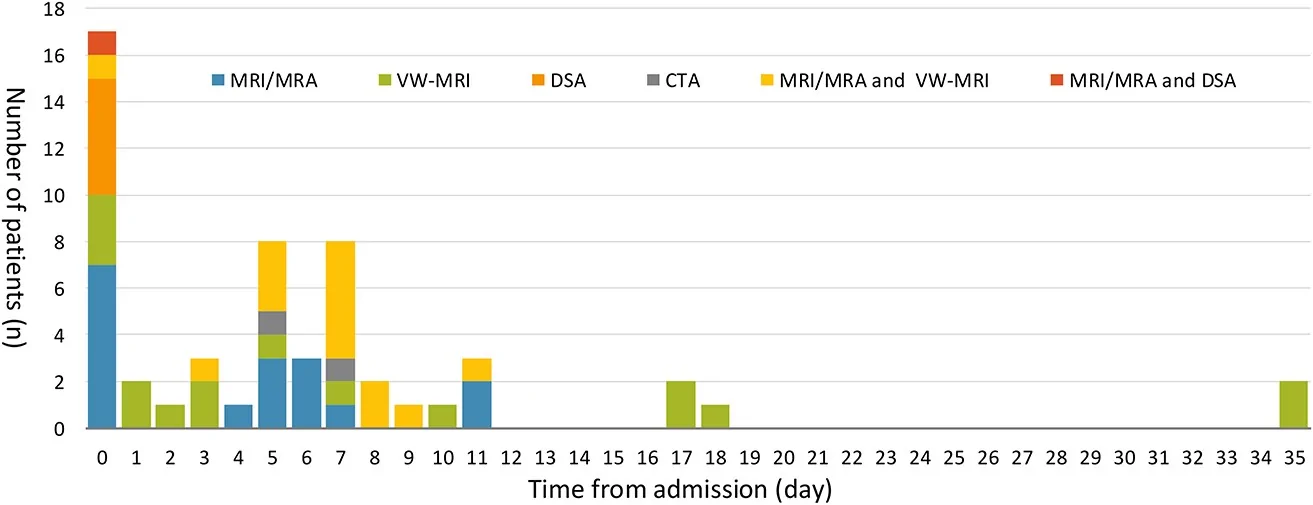
Time from admission to diagnosis and imaging examinations that contributed to the diagnosis of IAD. Stacked bar graph showing the number of patients newly diagnosed with IAD on each day after admission. The horizontal axis indicates days from admission, and the vertical axis shows the number of patients diagnosed on each day. Within each bar, colour indicates the imaging examination or combination of examinations that fulfilled the diagnostic criteria on that day. Abbreviations: CTA = computed tomography angiography; DSA = digital subtraction angiography; IAD = intracranial artery dissection; MRA = magnetic resonance angiography; MRI = magnetic resonance imaging; VW-MRI = vessel wall MRI.

Figure 1 shows the number of patients newly diagnosed with IAD on each day after admission, grouped by the imaging examinations that first fulfilled the diagnostic criteria.

The diagnosis of IAD was made at a median of 5 days (IQR: 0–8) after admission and 6 days (IQR: 1–10) after symptom onset. Seventeen patients (32%) were diagnosed with IAD on the day of admission. Of the 53 patients with IAD, 77% had been diagnosed by Day 7, 91% by Day 14, and the latest diagnosis was made on Day 35.

Initial MRI/MRA fulfilled the diagnostic criteria for IAD in only 9 patients (17%), based on findings of intramural haematoma (*n* = 1), intimal flap or double lumen (*n* = 2) or pearl-and-string sign (*n* = 6). In the remaining 44 patients (83%), the initial MRI/MRA demonstrated nonspecific findings, including occlusion (*n* = 20), stenosis (*n* = 16), dilation (*n* = 4) or normal appearance (*n* = 4) of the affected artery. In these patients, the diagnosis of IAD was established using follow-up MRI/MRA, VW-MRI, DSA or CTA.

**Figure 2:**
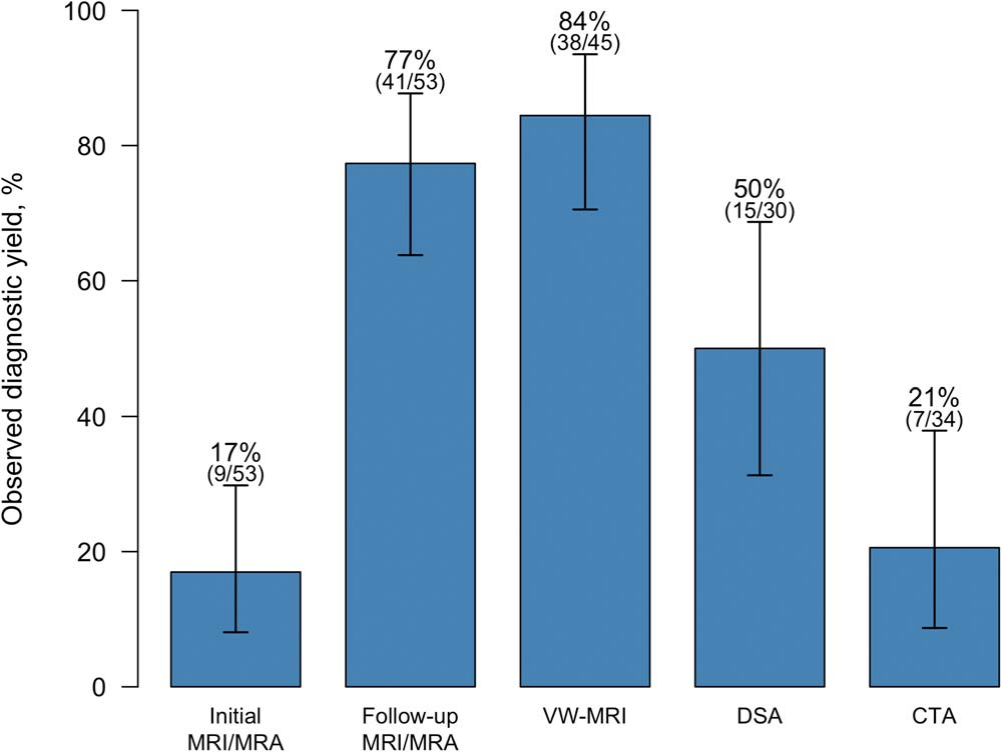
Observed diagnostic yield for intracranial artery dissection (IAD) among patients who underwent each examination. Numbers above each bar indicate the proportion and corresponding numerator/denominator (*n*/*N*), where n is the number of patients whose imaging findings fulfilled the diagnostic criteria and *N* is the number of patients who underwent each examination. Error bars represent 95% CIs calculated using the Clopper–Pearson exact method. Abbreviations: CTA = computed tomography angiography; DSA = digital subtraction angiography; IAD = intracranial artery dissection; MRA = magnetic resonance angiography; MRI = magnetic resonance imaging; VW-MRI = vessel wall MRI.

The observed diagnostic yield was 17% (9 of 53; 95% CI, 8–30) for initial MRI/MRA, 77% (41 of 53; 95% CI: 64–88) for follow-up MRI/MRA, 84% (38 of 45; 95% CI: 71–94) for VW-MRI, 50% (15 of 30; 95% CI: 31–69) for DSA and 21% (7 of 34; 95% CI: 9–38) for CTA (Figure 2). Because the examinations were not performed uniformly, additional cohort-level diagnostic contributions were calculated using the whole IAD cohort (*n* = 53) as the denominator; these values are provided in Table S3. In the sensitivity analysis restricted to definite IAD (*n* = 43), the principal findings were consistent with those of the full cohort. Initial MRI/MRA identified IAD in 7 patients (16%; 95% CI, 7–31), and additional cases were diagnosed by subsequent imaging (Table S3).

Among the patients who underwent each examination, the most common diagnostic findings were rapid changes in morphology on follow-up MRI/MRA (70%, 37 of 53), intramural haematoma on VW-MRI (69%, 31 of 45) and pearl-and-string sign on DSA (43%, 13 of 30) and CTA (18%, 6 of 34) (Table 3). In the 8 patients who did not undergo VW-MRI, the diagnosis was made on the initial MRI/MRA in 2 patients and on DSA in 1 (both at admission), and on follow-up MRI/MRA in the remaining 5; the median time to diagnosis was 7 days after onset (5 days after admission).

**Table 3:** Radiological findings.

	MRI/MRA ^a^ (*n* = 53)	VW-MRI (*n* = 45)	DSA (*n* = 30)	CTA (*n* = 34)
**Intramural haematoma**	5 (9)	31 (69)	NA	NA
**Intimal flap or double lumen**	6 (11)	6 (13)	3 (10)	1 (3)
**Stenosis or occlusion of intracranial artery, secondary development towards dilation**	6 (11)	3 (7)	0	0
**Pearl and string sign**	21 (40)	4 (9)	13 (43)	6 (18)
**Rapid change in morphology**	37 (70)	NA	NA	NA
**Aneurysmal dilation**	20 (38)	12 (27)	11 (37)	7 (21)
**Stenosis**	35 (66)	NA	23 (77)	17 (50)
**Occlusion**	22 (42)	NA	6 (20)	11 (32)

Data are presented as n (%).

Abbreviations: CTA = computed tomography angiography; DSA = digital subtraction angiography; MRA = magnetic resonance angiography; MRI = magnetic resonance imaging; NA = not evaluated; VW-MRI = vessel wall MR imaging.

^a^Findings on initial or follow-up MRI/MRA.

**Figure 3:**
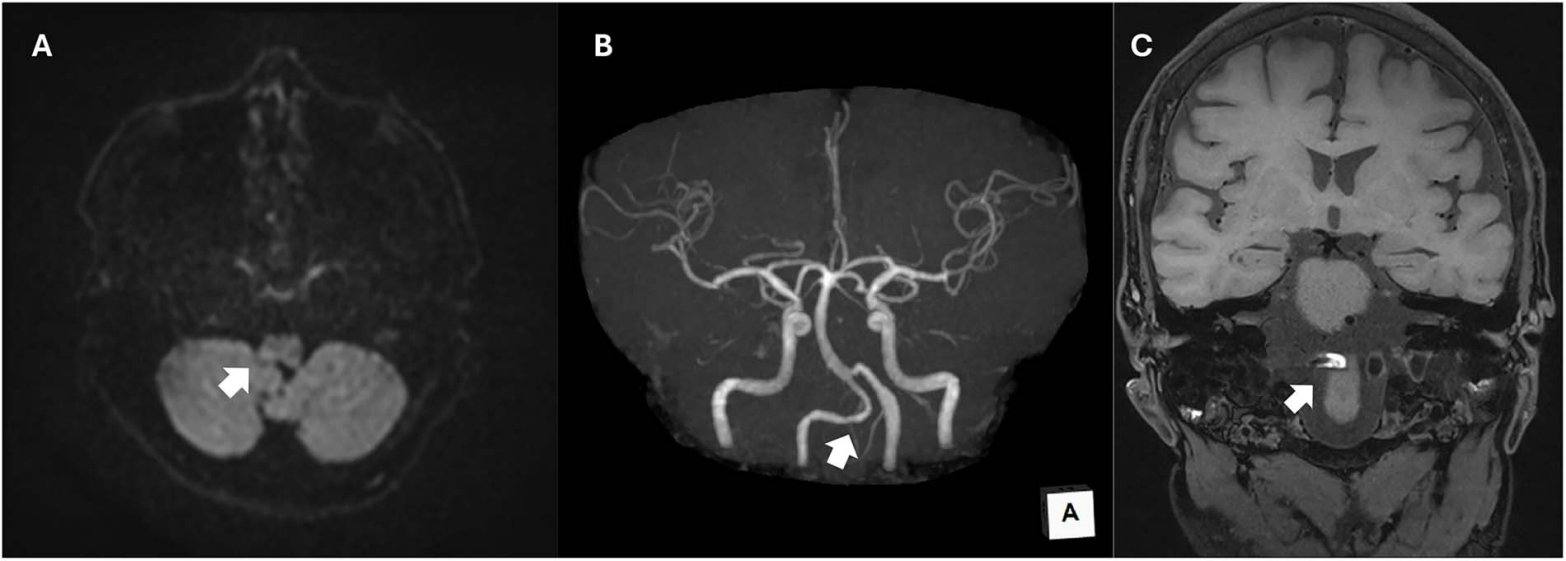
Representative case of IAD involving a patient with medullary infarction. (A) on day 0 (day of admission), DWI demonstrated an acute infarction in the right lateral medulla oblongata (white arrow, A). (B) on day 0, 3D time-of-flight MRA showed focal stenosis of the affected right vertebral artery, without definitive evidence of dissection (white arrow, B). (C) on day 8, VW-MRI demonstrated an intramural haematoma in the right intracranial vertebral artery (white arrow, C). Abbreviations: DWI = diffusion-weighted imaging; IAD = intracranial artery dissection; MRA = magnetic resonance angiography; VW-MRI = vessel wall MRI.

A representative case is shown in Figure 3. Initial MRI/MRA on Day 0 showed only focal stenosis of the right intracranial VA, a nonspecific finding without definite features of dissection. However, VW-MRI performed on Day 8 revealed an intramural haematoma in the right intracranial VA, establishing the diagnosis of IAD.

## Discussion

In this study of young adults with ischaemic stroke who ultimately met the imaging criteria for IAD, initial routine MRI/MRA alone was insufficient to establish the diagnosis in most cases. Although all patients underwent MRI/MRA at admission, imaging findings fulfilling the diagnostic criteria for IAD were identified in only 17% of patients at the initial evaluation. The diagnosis was often established later: the median time to diagnosis was 5 days after admission; 77% of patients were diagnosed by Day 7, and the latest diagnosis was made on Day 35. This temporal pattern underscores the critical role of follow-up and supplemental imaging in the diagnostic process.

Our findings highlight a limitation of initial routine MRI/MRA in the early diagnosis of IAD. Characteristic imaging features of dissection, such as intramural haematoma, intimal flap and double lumen, are often subtle or absent in the acute phase.^8^ This is likely due to the dynamic evolution of dissection, whose characteristic features emerge over the following days and may not be captured on a single early imaging study. As a result, reliance on initial MRI/MRA study, including intracranial 3D TOF-MRA, may lead to delayed or missed diagnoses. Other MR-based vascular imaging techniques, such as contrast-enhanced MRA, may better delineate the vascular lumen in selected settings, but they require contrast administration. These techniques were not included in our routine baseline protocol.

Importantly, our study suggested that the diagnostic process for IAD may benefit from a stepwise, sequential imaging approach. In our cohort, the median time to diagnosis was 5 days after admission, being shorter than that reported in previous studies,^15^ likely owing to the combined use of further imaging, particularly VW-MRI, and routine follow-up MRI/MRA performed approximately one week after admission. While scheduled follow-up MRI/MRA served as an effective safeguard against missed diagnoses, a marked proportion of cases were diagnosed earlier through the flexible use of additional imaging examinations at the discretion of treating physicians. Notably, several patients were not diagnosed until more than 2 weeks after symptom onset, with the latest diagnosis made on Day 35, reflecting the evolving nature of IAD.

VW-MRI was additionally performed at the discretion of the treating physicians in 45 of 53 patients with IAD, and diagnostic findings were identified in 38 of these patients (84%). Its ability to enable direct visualisation of vessel wall pathology facilitates the detection of dissection-related changes that are not apparent on conventional lumen-based imaging.^16^ Accumulating evidence suggests that VW-MRI is useful for differentiating arterial dissection from other intracranial arterial diseases.^17^ A more recent study reporting histopathological correlation further supports its diagnostic validity.^18^ In line with these reports, our findings suggest that VW-MRI may provide useful diagnostic information in selected patients with suspected IAD, particularly when initial MRI/MRA findings are inconclusive. However, because VW-MRI was performed selectively and at variable time points, this observed proportion should not be interpreted as comparative diagnostic accuracy or evidence of superiority over other imaging modalities.

DSA and CTA served as complementary diagnostic tools in selected cases. These lumen-based modalities can provide clinically important information on vascular morphology, particularly when detailed vascular anatomy is required or when MRI is unavailable or contraindicated. In this study, DSA and CTA were often performed early after onset, when luminal changes related to dissection may not yet have become apparent. Therefore, their observed detection rates should be interpreted in light of patient selection and examination timing, rather than as a basis for direct comparison across modalities.

Several clinical features and infarction patterns at presentation may also raise suspicion of IAD. In our cohort, patients with IAD were more likely to present with headache or neck pain, as well as posterior circulation infarction, particularly involving the medulla oblongata. Recognition of these features may help clinicians maintain a high index of suspicion for IAD, even when initial imaging findings are inconclusive.

This study has several limitations. First, as a secondary analysis of a prospective cohort, the present study was not specifically designed to test prespecified hypotheses regarding imaging strategies for IAD, which may have limited the scope of the analyses. Second, the number of patients with IAD was relatively small, and the study population consisted exclusively of Japanese patients. Therefore, caution is warranted when generalising our findings to other ethnic groups or healthcare settings. Third, the imaging protocol was not fully standardised: VW-MRI, DSA and CTA were performed only in selected patients at different time points, at the discretion of the treating physicians according to clinical suspicion and clinical course, including worsening headache or neurological symptoms. This may have introduced verification bias. Therefore, the observed diagnostic yields reflect the proportion of patients whose imaging findings fulfilled the diagnostic criteria among those who underwent each examination, rather than the sensitivity or comparative diagnostic accuracy of each modality. Fourth, the final diagnosis relied on imaging features that were also used to judge whether each examination met the diagnostic criteria, and these assessments were not blinded. Therefore, the results are subject to incorporation bias and should not be interpreted as independent estimates of diagnostic accuracy. Lastly, because IAD was defined by imaging features, cases lacking characteristic imaging findings may have harboured undiagnosed IAD. The true number of IAD cases may therefore have been underestimated, and our analysis was restricted to patients who fulfilled the imaging diagnostic criteria for IAD.

In conclusion, among young adults with ischaemic stroke who ultimately met the imaging criteria for IAD, initial MRI/MRA alone did not fulfil these criteria in most cases, and the diagnosis was often established by subsequent imaging. Our findings suggest that repeated and multimodal imaging may contribute to the detection of IAD when it is suspected despite inconclusive initial imaging. Whether a structured, time-dependent diagnostic protocol would further improve the timely diagnosis of IAD warrants prospective evaluation.

## Supplementary Material

Revised_Clean_Supplementary_Materials_aakag115

## Data Availability

The data that support the findings of this study are not publicly available as they contain information that could compromise the privacy of research participants; however, they are available from the corresponding author (N.M.) upon reasonable request.
