# Supplementary material for: Initial magnetic resonance imaging/magnetic resonance angiography may miss intracranial artery dissection in young adults with stroke: the role of subsequent imaging
Source: Eur Stroke J. 2026 Sep 18;11(9):aakag115. doi: 10.1093/esj/aakag115 (PMC13588095; doi:10.1093/esj/aakag115)
Supplement: Supplementary file 1 [file revised_clean_supplementary_materials_aakag115.docx]

**Supplementary Materials**

Table S1. Definition and grading of imaging diagnostic criteria as evidence of IAD ^1^

**Imaging diagnostic criteria of IAD**

At least one of the three following features should be present when diagnosing IAD

・Irregular aneurysmal dilation at a non-branching site of an intracranial artery, with at least one of the following criteria

・Intramural hematoma or double lumen

・Rapid change in morphology on repeating imaging (increase or reduction in size, subsequent appearance of stenosis)

・pearl-and-string sign

・Long irregular stenosis of an intracranial artery, with at least one of the following criteria

・Intramural hematoma or double lumen

・Rapid change in morphology on repeating imaging (increase or reduction in size, subsequent appearance of stenosis)

・pearl-and-string sign

・Occlusion of an intracranial artery that recanalizes in irregular aneurysmal dilation at a non-branching site, or irregular stenosis

**Grading of imaging diagnostic criteria as evidence of IAD**

Definite IAD

・Stenosis or occlusion of an intracranial artery secondarily developing towards irregular aneurysmal dilation at a non-branching site

・Intramural hematoma, or double lumen

・Pathological confirmation of intracranial artery dissection

Probable IAD

・Pearl-and-string sign without subarachnoid hemorrhage, or still present >1 month after subarachnoid hemorrhage

・Irregular aneurysmal dilation at non-branching site with rapid change in morphology (Increase or reduction in size, or subsequent appearance of stenosis)

Possible IAD

・Irregular aneurysmal dilation at non-branching site without change in morphology on repeated imaging within 6-12 months after first imaging

・Irregular stenosis of an intracranial artery, with reduction in size or disappearance over time

IAD: intracranial artery dissection.

Table S2. Stroke etiology in the whole cohort (N = 275).

| Stroke etiology |  |
| --- | --- |
| Large artery atherosclerosis | 23 (8) |
| Cardioembolism | 17 (6) |
| Small vessel occlusion | 72 (26) |
| Other determined cause | 91 (33) |
| IAD | 53 (19) |
| EAD | 15 (5) |
| Other causes | 23 (8) |
| Undetermined cause | 72 (26) |

Data are presented as n (%). Stroke etiology according to the TOAST criteria ^2^ based on the protocol-based workup, as described previously ^3^. Other causes comprised moyamoya disease (n = 7), vasculitis (n = 5), cancer-associated thrombosis (n = 4), carotid web (n = 2), reversible cerebral vasoconstriction syndrome (n = 1), bow hunter syndrome (n = 1), protein S deficiency (n = 1), heparin-induced thrombocytopenia (n = 1), and essential thrombocytosis (n = 1).

EAD: extracranial artery dissection, IAD: intracranial artery dissection, TOAST: Trial of Org 10172 in Acute Stroke Treatment.

Table S3. Proportion of patients with intracranial artery dissection (IAD) meeting the imaging diagnostic criteria for each examination, in the full cohort and in the subgroup of definite IAD.

|  | Full cohort (n = 53) | | Definite IAD (n = 43) | |
| --- | --- | --- | --- | --- |
|  | Patients examined | Whole-cohort | Patients examined | Whole-cohort |
| Initial MRI/MRA | 9/53 (17 [8–30]) | 9/53 (17 [8–30]) | 7/43 (16 [7–31]) | 7/43 (16 [7–31]) |
| Follow-up MRI/MRA | 41/53 (77 [64–88]) | 41/53 (77 [64–88]) | 32/43 (74 [59–87]) | 32/43 (74 [59–87]) |
| VW-MRI | 38/45 (84 [71–94]) | 38/53 (72 [58–83]) | 37/39 (95 [83–99]) | 37/43 (86 [72–95]) |
| DSA | 15/30 (50 [31–69]) | 15/53 (28 [17–42]) | 11/25 (44 [24–65]) | 11/43 (26 [14–41]) |
| CTA | 7/34 (21 [9–38]) | 7/53 (13 [6–25]) | 5/27 (19 [6–38]) | 5/43 (12 [4–25]) |

Values are shown as n/N (proportion % [95% Clopper–Pearson exact confidence interval]). “Patients examined” refers to the patients who underwent each examination; “whole-cohort” refers to all patients with IAD (full cohort, n = 53) or all patients with definite IAD (n = 43). Initial and follow-up MRI/MRA were performed in all patients, so the two denominators are identical for these examinations.

CTA: computed tomography angiography, DSA: digital subtraction angiography. IAD: intracranial artery dissection, MRA: magnetic resonance angiography, MRI: magnetic resonance imaging, VW-MRI: vessel wall MRI.

**Reference**

1.	Debette S, Compter A, Labeyrie MA, et al. Epidemiology, pathophysiology, diagnosis, and management of intracranial artery dissection. Lancet Neurol 2015;14:640–654.

2.	Adams HP, Jr., Bendixen BH, Kappelle LJ, et al. Classification of subtype of acute ischemic stroke. Definitions for use in a multicenter clinical trial. TOAST. Trial of Org 10172 in Acute Stroke Treatment. Stroke 1993;24:35–41.

3.	Ohara T, Makita N, Fujinami J, et al. Clinical Characteristics of Ischemic Stroke in Japanese Young Adults. Cerebrovasc Dis Extra 2025;15:154–161.
